# Effects of extruded pet foods containing dried yeast (*Saccharomyces cerevisiae*) on palatability, nutrient digestibility, and fecal quality in dogs and cats

**DOI:** 10.1093/tas/txad107

**Published:** 2023-09-06

**Authors:** Gary M Davenport, Stephanie S Block, Jennifer L Adolphe

**Affiliations:** ADM, Decatur, IL62526, USA; ADM, Decatur, IL62526, USA; ADM, Decatur, IL62526, USA

**Keywords:** cat, dog, digestibility, protein, palatability, yeast

## Abstract

Global protein shortages and sustainability concerns have increased consumer demand for non-animal-derived protein. Dried whole-cell yeast (*Saccharomyces cerevisiae*) may be a suitable alternative to rendered protein meals in pet foods. The objective of this study was to investigate the effects of dried yeast in dog and cat foods on indicators that pet parents typically use to evaluate the suitability of a food for their pet. For this evaluation, two dog and two cat dry extruded diets were formulated. For each species, the test diet contained 10% dried yeast (Yeast) and the control diet was devoid of yeast (Control). Palatability, apparent total tract nutrient digestibility, and fecal quality of the foods were assessed in dogs and cats. Urine pH and specific gravity were measured in cats as indicators of urinary tract health. In dogs, the Yeast diet showed equivalent or better palatability compared to the Control diet based on total food consumption (*P* = 0.06), average daily consumption (day 1, *P* = 0.10; day 2, *P* = 0.54), and first choice preference over 2 consecutive days (*P* = 0.005). Cats showed a strong preference for the Yeast diet with more than double the consumption during the 2-d test period (*P* = 0.001). More cats showed a first-choice preference for the Yeast diet (24 vs. 16), but the difference was not statistically significant (*P* = 0.21). There were no significant differences in stool quality or nutrient digestibility when fed Yeast vs. Control diets to the dogs and cats (*P* > 0.05). All cats produced urine with pH and specific gravity values within the normal range, though specific gravity was lower in the Control group (*P* = 0.003). This study provides support for the acceptability and digestibility of dog and cat diets containing dried yeast as an alternative protein source.

## Introduction

Novel protein sources have emerged as an important trend in the pet food industry in response to consumer demand, sourcing constraints, and sustainability concerns. Dried whole-cell yeast (*S. cerevisiae*) is an alternative to traditional protein sources (i.e., animal-derived protein) that aligns with this trend and has been shown to have beneficial health effects in several animal species, including the modulation of the colonic microbiota in dogs (Moyad et al., 2008; Stercova et al., 2016; Lin et al., 2019).

Limited research has been conducted with regard to the effects of dried yeast on the palatability, digestibility, stool quality, and urinalysis when used as a principle source of protein in nutritionally complete and balanced foods for dogs and cats. To date, most research has focused on low inclusion levels of whole yeast or yeast components such as yeast cell walls, beta-glucans, or mannan-oligosaccharides (Grieshop et al., 2004; Kroll et al., 2020; Lin et al., 2020; Van den Abbeele et al., 2020; Matheus et al., 2021). However, whole yeast offers numerous nutritional advantages in addition to these functional benefits. For example, dried yeast has been demonstrated to be a highly digestible source of protein and essential amino acids as measured using the precision-fed cecectomized rooster assay, a surrogate model for dogs and cats to determine amino acid digestibility and protein quality (Reilly et al., 2020). However, further investigation is necessary to determine the acceptability and digestibility of dried yeast when used at moderate levels in pet foods.

The objective of the current study was to investigate dried yeast as a protein source in dog and cat foods using commercially relevant inclusion levels (10%) to examine key food attributes that pet owners may consider when choosing a food for their dog or cat, such as palatability, digestibility, and stool quality. Since urinary tract health is a common concern in cats, the effects of dried yeast on urine pH and specific gravity were also assessed. The hypothesis was that the dried yeast dog and cat foods would be equivalent or better than the control foods without dried yeast when evaluated using standard nutritional end-points.

## Materials and Methods

All studies were conducted at Summit Ridge Farms (Susquehanna, PA), a United States Department of Agriculture-licensed facility (no. 23-R-0126) under the Animal Welfare Act. The Summit Ridge Farms Ethics Committee approved all animal care protocols.

### Experimental Diets

Two dog and two cat dry extruded kibble diets were formulated to meet the Association of American Feed Control Officials (AAFCO) nutrient profiles for maintenance of adult dogs and adult cats, respectively (Association of American Feed Control Officials, 2016). For each species, the Yeast diet included Versity dried yeast (ADM Animal Nutrition, Decatur, IL, USA) at 10% inclusion and the Control diet did not contain a source of dried yeast. The nutrient composition of the dried yeast is shown in Table 1. For the Yeast diet, dried yeast was included at the expense of pork meal and wheat midds to provide similar nutrient content in the Yeast and Control diets. The ingredient composition of the Control and Yeast diets for dogs and cats is shown in Table 2.

**Table 1.** Analyzed nutrient composition of dried yeast

Nutrient	Composition
Moisture, %	7.4
Crude protein, % DM	51.8
Crude fat, % DM	3.3
Ash, % DM	2.7
Crude fiber, % DM	6.6

DM, dry matter.

**Table 2.** Ingredient composition of Control and Yeast diets fed to dogs and cats

Ingredient, %	Dog		Cat	
	Control	Yeast	Control	Yeast
Chicken meal	13.1	13.1	20.0	20.0
Brewers rice	13.0	13.0	12.0	12.0
Corn	13.0	13.0	12.0	12.0
Wheat grain	13.0	13.0	12.0	12.0
Corn gluten meal	10.0	10.0	10.0	10.0
Versity dried yeast	—	10.0	—	10.0
Pork meal	9.9	2.2	9.2	1.3
Wheat midds	8.7	2.6	7.4	1.9
Poultry fat	9.4	10.1	9.5	10.2
Beet pulp, palatant, flaxseed, calcium propionate, ethoxyquin	6.2	6.2	6.2	6.2
Potassium, salt, calcium carbonate, monocalcium phosphate	2.9	6.0	1.0	3.6
Vitamins and trace minerals^1^	0.8	0.8	0.7	0.8

^1^Choline chloride, iron sulfate, zinc sulfate, vitamin E, zinc oxide, manganese sulfate, copper sulfate, selenium, niacin, biotin, calcium pantothenate, riboflavin, vitamin A, menadione sodium bisulfite complex, thiamin mononitrate, vitamin B12, calcium iodate, pyridoxine HCl, vitamin D3, cobalt carbonate, folic acid.

The Yeast and Control diets were blended and manufactured under the same conditions and locations (ADM Animal Nutrition, Effingham, IL in partnership with a contracted third-party U.S. pet food pilot plant facility). Prior to extrusion, diets were uniformly ground and screened, and subsequently processed using a preconditioner and single-screw extruder. Immediately after drying, dog and cat kibbles were sprayed with poultry fat along with commercially available dry (6C2AQ or F23004, respectively) and liquid (C14074 and LC657, respectively) hydrolyzed protein palatants from AFB International (St. Charles, MO, USA). Kibble dimensions (diameter × thickness) and density were 10.2 × 6.1 mm and 304 g/L for the dog Yeast diet; 10.2 × 5.1 mm and 336 g/L for the dog Control diet; 8.1 × 4.3 mm and 288 g/L for the cat Yeast diet; and 7.6 × 3.8 mm and 400 g/L for the cat Control diet, respectively.

Nutrient analyses of the dried yeast were performed at the University of Missouri using the following methods: moisture (AOAC 930.15), crude protein (AOAC 990.03), crude fat (AOAC 920.39), crude fiber (AOAC 962.09, AOCS Ba6-84), and ash (AOAC 942.05). Nutrient analyses of the diets were completed at Eurofins (Des Moines, IA) using accepted laboratory methods for energy (bomb calorimetry), moisture (AOAC 925.09), crude protein (AOAC 992.15, AOAC 990.03, AOCS Ba 4e-93), acid-hydrolyzed fat (AOAC 954.02), crude fiber (AOAC 962.09, AOCS Ba 6-84), ash (AOAC 942.05), calcium (AOAC 984.27 mod, 927.02 mod, 985.01 mod, and 965.17 mod), and phosphorus (AOAC 984.27 mod, 927.02 mod, 985.01 mod, and 965.17 mod). Nitrogen-free extract (NFE) was calculated as 100−(%crude protein+% crude fat+% crude fiber+% ash) (Association of American Feed Control Officials, 2016). Metabolizable energy (ME) was calculated using the modified Atwater equation (Association of American Feed Control Officials, 2016): ME=10×[(3.5×%Protein)+(8.5×%Fat)+(3.5×%NFE)].

### Palatability

Two-day, two-bowl palatability trials were conducted as described by Griffin (2003) using 20 beagle dogs (14 intact males and 6 intact females; age 7.4 ± 0.8 yr) and 20 domestic shorthair cats (8 neutered males and 12 spayed females; age 10.1 ± 0.8 yr; body weight 5.0 ± 0.2 kg). Once daily, animals were offered the Yeast and Control diets in two stainless steel bowls, each containing 400 g of diet for dogs and 65 g of diet for cats. Bowl placement was reversed on the second day and both bowls were presented for 30 min. If one diet was completely consumed prior to the end of 30 min, both bowls were removed. Total food consumption and first choice preference were recorded for each animal and intake ratios for each diet were calculated.

### Digestibility and Stool Quality

Apparent total tract digestibility (ATTD) of dietary nutrients was assessed using a completely randomized study design and the AAFCO dog and cat food ME quantitative collection protocol (Association of American Feed Control Officials, 2016). Twelve beagle dogs (eight intact males and four intact females; 8 ± 1 yr; body weight 11.2 ± 0.6 kg; body condition score 3.1 ± 0.1 on a 5-point scale) and 14 domestic shorthair cats (six neutered males, two intact females, and six spayed females; age 7 ± 0.9 yr; weight 4.5 ± 0.2 kg; body condition score 3.9 ± 0.1 on a 5-point scale) participated in the study. During the study, animals were housed individually in kennels (1.44 m^2^ for dogs; 0.76 m^2^ for cats) with a 12 h light/12 h dark schedule and temperature maintained within 10 to 30 °C. Fresh water was available at all times.

Individual dogs and cats were randomly divided into two groups (dogs *n* = 6/group; cats *n* = 7/group) with each group then randomly assigned to receive either the Yeast or Control diet. The 10-d test period consisted of a 5-d acclimation period followed by 5-d of fecal collection. The Yeast or Control diet was the sole source of food for the duration of the testing period. Animals were fed once daily at the same time each day and food consumption was recorded daily. Food intake was individually calculated based on ME requirements. Body weight was measured daily during the acclimation period, on day 1 of the collection period, and on the last day of the collection period. Food intake was only adjusted accordingly during the acclimation period to maintain body weight, if necessary.

Feces were collected a minimum of three times daily or as often as needed during the collection period to ensure clean samples for each dog. All fecal samples were assessed for fecal quality using the following fecal scoring: 1.0 = watery diarrhea; 1.5 = diarrhea; 2.0 = moist, no form; 2.5 = moist, some form; 3.0 = moist, formed; 3.5 = well-formed, sticky; 4.0 = well-formed; 4.5 = hard, dry; 5.0 = hard, dry, crumbly. A fecal score of 3.5 was considered to be ideal. After scoring, feces were collected in individual bags, weighed, and stored at −20 °C until nutrient analyses for ATTD determination. Nutrient content of the feces was analyzed at Eurofins using the same methods as described previously for the diets except for moisture (AOAC 925.09). Nutrient ATTD (%) was calculated using the equation:


$$\begin{array}{l} ATTD=\frac{\begin{array}{c} \left[\left(Total\;Food\;Consumed\right)\times \;\left(\%\;Nutrient\;in\;Food\right)]-\;[\left(Total\;Weight\;of\;Stool\right)\times \;\left(\%\;Nutrient\;in\;Stool\right)\right] \end{array}}{\begin{array}{c} \left(Total\;Food\;Consumed\right)\times \;(\%\;Nutrient\;in\;Food) \end{array}} \end{array}$$

Dietary ME (kcal/g) was also calculated using digestible energy and protein values and the AAFCO (2016) equation:


$$\begin{array}{l} ME=\frac{\begin{array}{c} \left[(Gross\;Energy\;in\;Food-\;Gross\;Energy\;in\;Stool)\times \;(Grams\;Protein\;Digested\times \;Urine\;Correction\;Factor)\right] \end{array}}{(Amount\;of\;Food\;Consumed)}. \end{array}$$

The corresponding correction factor for energy lost in the urine was 1.25 kcal/g for dogs and 0.86 kcal/g for cats.

### Cat Urinalysis

A separate study in cats assessed the effect of the Yeast and Control diets on urine pH and specific gravity. Twenty domesticated short hair cats (8 neutered males, 10 spayed females, and 2 intact females; age 4.8 ± 0.5; body weight 4.2 ± 0.2 kg; body condition score 3.3 ± 0.1 on a 5-point scale) were individually housed and divided into two groups (*n* = 10 per group) that received either the Yeast or Control diet for 7 d. Food was offered at the same time each day and was provided over a 20-h period from 11:00 a.m. until 7:00 a.m. the next day. Daily food consumption was recorded for each cat. Body weights were recorded on days 1, 3, 5, and 7.

On the seventh day of the trial, each cat had access to a clean, empty litter pan to obtain free-catch urine voids. For cats that did not void in the litter pan, urine samples were obtained via cystocentesis. Samples were immediately analyzed onsite using a calibrated pH meter and specific gravity refractometer.

### Statistical Analyses

Analyses were performed using Microsoft Excel (Redmond, WA, USA) and SAS (SAS Institute Inc., Cary, NC). Data were expressed as means ± SEM. For the palatability data, Chi-square analysis was used to determine preference by first choice and total food consumption was analyzed using a Wilcoxon signed rank test. Independent *t*-tests were used to analyze data from the digestibility trials and urinalysis. Differences were considered significant at *P* < 0.05 with *P* ≤ 0.10 considered a trend.

## Results

The high protein content (52.4%) of the dried yeast made it a suitable replacement for pork meal to produce the Yeast test diets for both dogs and cats with similar nutrient compositions to the Control diets, as shown in Table 3.

**Table 3.** Nutrient composition of Control and Yeast diets fed to dogs and cats

Nutrient	Dog		Cat	
	Control	Yeast	Control	Yeast
Moisture, %	5.0	5.3	7.9	6.0
Crude protein, % DM	30.4	30.4	32.8	33.1
AHF, % DM	15.9	15.3	16.3	16.9
Crude fiber, % DM	2.3	1.8	2.1	1.6
Ash, % DM	8.9	10.0	8.2	8.2
Calcium, % DM	2.1	2.1	1.8	1.7
Phosphorus, % DM	1.5	1.5	1.2	1.1
NFE, % DM	42.4	42.5	40.7	40.2
GE, kcal/g DM	4.7	5.0	5.2	5.1
ME, kcal/g DM^1^	3.9	3.9	4.0	4.0

^1^Atwater calculation: ME = 10 × [(3.5 × % protein) + (8.5 × % Fat) + (3.5 × % NFE)].

DM, dry matter; AHF, acid-hydrolyzed fat; NFE, nitrogen-free extract; GE, gross energy; ME, metabolizable energy.

### Palatability

#### Dogs.

**Figure 1.**
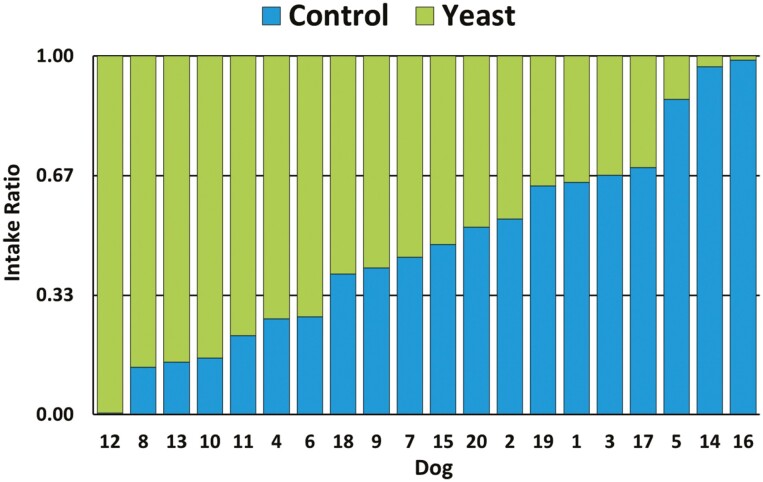
Individual intake ratios in dogs based on total consumption of Yeast diet vs. Control diet over a 2-d period (*n* = 20).

The total amount of food consumed by the 20 dogs during the 2-d trial was 6,953 g of the Yeast diet and 6,085 g of the Control diet resulting in a consumption ratio of 1.14:1 (*P* = 0.50). An intake ratio ≥ 0.67 indicates a 2:1 consumption ratio and represents a strong preference for one diet over the other. Thus, the Yeast diet was clearly preferred by seven dogs while five dogs clearly preferred the Control diet (Figure 1). Based on average daily consumption, there was a trend (*P* = 0.10) for dogs to consume more Yeast diet on day 1 (212.8 ± 26.3 g) vs. the Control diet (137.8 ± 21.1 g), while diet intakes were not different (*P* = 0.54) on day 2 for Yeast (134.9 ± 25.5 g) and Control (166.5 ± 26.5 g). Over both test days, the Control and Yeast diets were chosen first on 14 and 26 occasions, respectively, demonstrating a trend (*P* = 0.06) in the preference for the Yeast diet. The Yeast diet was chosen first on both days by significantly (*P* = 0.005) more dogs (*n* = 11) than the Control diet (*n* = 5).

#### Cats.

**Figure 2.**
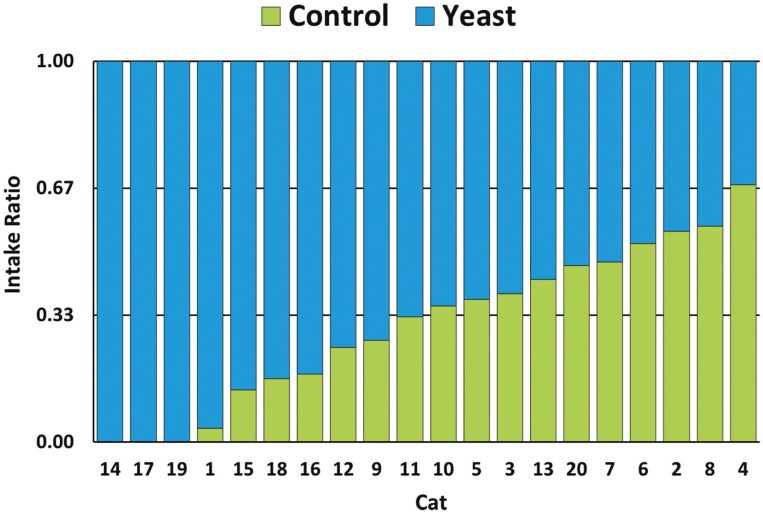
Individual intake ratios in cats based on total consumption of Yeast diet vs. Control diet over a 2-d period (*n* = 20).

Total diet consumption over the 2-d test period was significantly (*P* = 0.001) higher for the Yeast diet (1,778 g) compared to the Control (851 g) resulting in a consumption ratio of 2.09:1. This difference was due to cats consuming significantly (*P* = 0.001) more of the Yeast diet (47.5 ± 4.5 g) compared to the Control diet (14.2 ± 3.0 g) on day 2. Cats also consumed more Yeast diet (41.4 ± 4.8 g) than Control diet (28.4 ± 6.4 g) on day 1, but the difference was not significant (*P* = 0.19). Based on intake ratios ≥ 0.67, the Yeast diet was clearly preferred by 10 cats, whereas only one cat showed a clear preference for the Control diet (Figure 2). Over both days, the Yeast diet was selected first on 24 occasions while the Control diet was selected first on 16 occasions resulting in a ratio of 1.5:1 (*P* = 0.21). Individually, six cats chose the Yeast diet first on both days while two cats chose the Control diet first (*P* = 0.30).

### Stool Quality and Nutrient Digestibility

#### Dogs.

During the 10-d digestibility trial, body weight increased by 0.53% ± 1.1% and 2.3% ± 0.8% for dogs fed the Yeast and Control diets, respectively (*P* = 0.40). Total 10-d food consumption was similar (*P* = 0.50) for the Yeast diet (1,329 ± 45.5 g) and Control diet (1,236 ± 85.1 g). Total stool weight during the 5 d of collection was also similar (*P* = 0.15) for dogs fed the Yeast diet (620 ± 72 g) and the Control diet (408 ± 57 g). Stool quality scores were similar (*P* > 0.05) between the Yeast (3.38 ± 0.05) and Control (3.34 ± 0.05) diets (Table 4). The percentage of stools rated as highly acceptable (3.0 to 4.0) were 96% for dogs fed the Yeast diet and 93% for dogs fed the Control diet. No significant differences (*P* > 0.05) in nutrient digestibility were observed between the Yeast and Control diets (Table 5). For the Yeast diet, ATTD values for dry matter, organic matter, protein, and energy ranged between 84.4% and 89.9% while the ATTD fat value was 93.7% and ATTD ash value was 49.6%. ATTD values for the Control diet ranged between 86.6% and 89.9% for dry matter, organic matter, and energy, while ATTD values for protein, fat, and ash were 90.8%, 95.2%, and 53.7%, respectively. The ME values calculated using digestible energy and protein were similar (*P* > 0.05) as the Yeast diet contained 4.02 ± 0.05 kcal/g and the Control diet contained 3.91 ± 0.05 kcal/g.

**Table 4.** Stool quality of Control and Yeast diets fed to dogs and cats

Stool score^1^	Dog		*P*-value	Cat		*P*-value
	Control	Yeast		Control	Yeast	
Mean score	3.33 ± 0.05^2^	3.38 ± 0.05	0.62	3.07 ± 0.08	3.13 ± 0.11	0.66

^1^Subjective scores: 1.0 = watery diarrhea; 1.5 = diarrhea; 2.0 = moist, no form; 2.5 = moist, some form; 3.0 = moist, formed; 3.5 = well-formed, sticky; 4.0 = well-formed; 4.5 = hard, dry; 5.0 = hard, dry, crumbly.

^2^Mean ± SEM.

**Table 5.** Apparent nutrient digestibility of Control and Yeast diets fed to dogs and cats

Nutrient	Dog		*P*-value	Cat		*P*-value
	Control	Yeast		Control	Yeast	
Dry matter, %	86.6 ± 1.9^1^	84.4 ± 2.1	0.46	80.6 ± 1.6	81.8 ± 1.3	0.56
Organic matter, %	89.8 ± 1.5	88.3 ± 1.6	0.48	84.6 ± 1.3	85.8 ± 1.1	0.45
Protein, %	90.8 ± 1.4	89.9 ± 1.5	0.67	84.1 ± 1.9	83.2 ± 1.5	0.69
Fat, %	95.2 ± 0.7	93.7 ± 0.8	0.20	86.2 ± 1.6	86.6 ± 1.3	0.84
Ash, %	53.7 ± 6.5	49.6 ± 7.1	0.68	36.6 ± 5.4	36.3 ± 4.2	0.96
Energy, %^2^	89.8 ± 1.4	89.0 ± 1.5	0.71	83.9 ± 1.7	85.5 ± 1.4	0.45
ME, kcal/g^3^	4.02 ± 0.05	3.91 ± 0.05	0.16	3.80 ± 0.06	3.96 ± 0.05	0.04

^1^Mean ± SEM.

^2^Digestible energy measured via bomb calorimetry.

^3^Metabolizable energy (ME) calculated using AAFCO (2016) protocol without urine collection.

#### Cats.

Over the 10-d digestibility trial, cats receiving the Yeast and Control diets lost 2.80% ± 1.3% and 1.96% ± 0.79% body weight, respectively (*P* = 0.41). Total 10-d food consumption was similar (*P* = 0.43) for the Yeast diet (250 ± 33 g) and Control diet (270 ± 22 g). Total stool weight during the 5 d of collection was also similar (*P* = 0.51) for cats fed the Yeast diet (155 ± 36 g) and Control diet (188 ± 29 g). There was no difference (*P* > 0.05) in stool quality for cats fed the Yeast (3.13 ± 0.11) and Control (3.07 ± 0.08) diets. No significant differences (*P* > 0.05) were observed for the nutrient ATTD values for cats fed the Yeast and Control diets as values ranged between 81.8% and 86.6% for dry matter, organic matter, protein, fat, and energy across both diets. When the ME content of each diet was calculated using digestible energy and protein, the ME content was higher (*P* = 0.04) for the Yeast diet (3.96 ± 0.05 kcal/g) compared with the Control diet (3.80 ± 0.06 kcal/g). Ash ATTD values were similar (*P* > 0.05) for the Yeast and Control diets (36.3% ± 4.2% and 36.6% ± 5.4%, respectively).

### Cat Urinalysis

**Figure 3.**
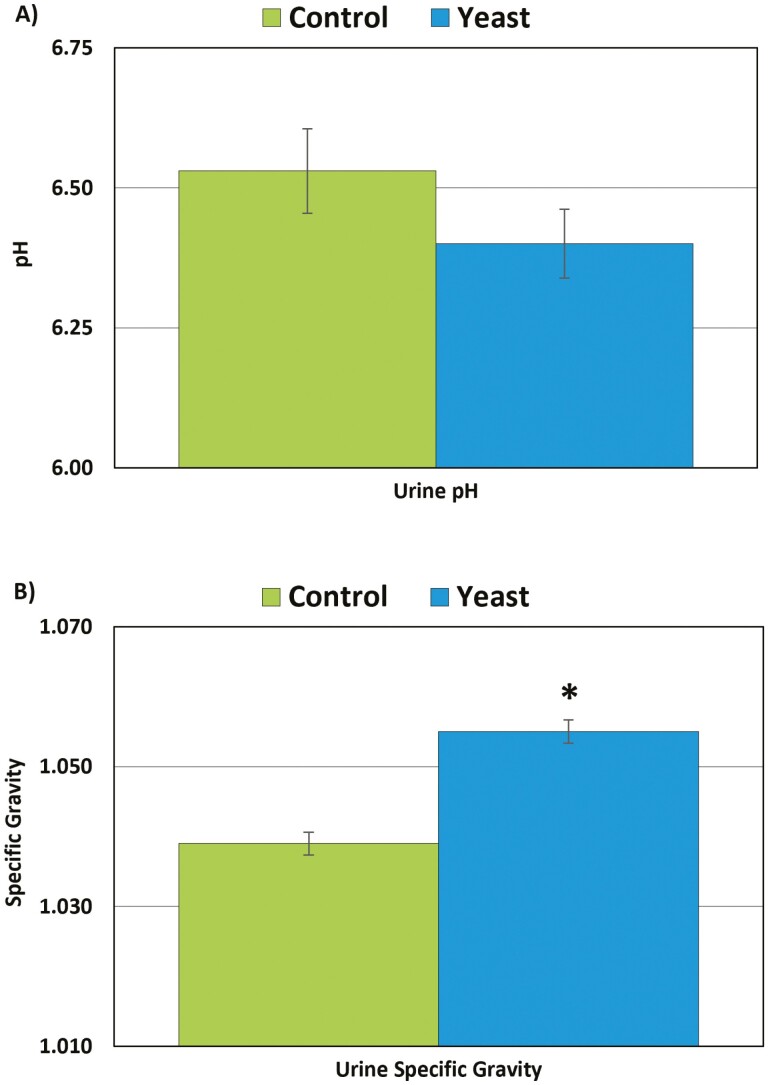
Urine pH (A) and specific gravity (B) in cats fed a Yeast or Control diet for 7 d (*n* = 10 per diet). *Urine specific gravity is significantly different for Yeast vs. Control (*P* < 0.05, independent *t*-test).

During the 7 d of the trial, daily diet consumption averaged 61 ± 3.2 g and 65 ± 1.1 g for the Yeast and Control diets, respectively. All cats produced urine within the normal physiological pH range (5.50 to 8.50) for healthy adult cats and no significant difference (*P* > 0.05) was observed between the two diets (Figure 3A). Specific gravity was also within the normal physiological range (1.001 to 1.080) for all cats (Figure 3B), but was lower (*P* = 0.003) for cats fed the Control diet (1.039 ± 0.0032) compared to the Yeast diet (1.055 ± 0.0033).

## Discussion

The results of this study demonstrated that dried whole yeast is a well-accepted, highly digestible source of protein for dogs and cats. Dried yeast inclusion at 10% did not compromise diet acceptability or utilization based on total food consumption or nutrient digestibility in dogs and cats. In addition, the diets with dried yeast resulted in the production of high-quality, well-formed stools by dogs and cats. This is one of the key visual indicators that pet owners use to assess the suitability of food for their pets. For these studies, isocaloric and isonitrogenous dog and cat foods were successfully formulated using 10% dried yeast as a replacement for pork meal and wheat midds, demonstrating its commercial viability as an alternative to animal-based protein ingredients.

In dogs, the Yeast diet showed equivalent or better palatability compared to the Control diet and cats demonstrated a clear preference for the Yeast diet. These results provide evidence contrary to the common perception that increasing levels of non-animal-based ingredients in pet foods contributes to reduced acceptance by dogs and cats. Since the perceived appeal of a pet food is another subjective measure used by pet owners to determine if a food is suitable for their dog or cat, pet food companies regularly assess the palatability of their products using a standard 2-d, two-bowl test. Thus, it is critical to ensure that the inclusion of novel ingredients in pet foods, such as dried yeast, does not negatively impact palatability. Although the Yeast diets used in the current study contained some meat ingredients, previous evidence indicates that meat may not be essential for enhancing product palatability for dogs and cats. Knight and Satchell (2021) reported that owners perceived plant-based pet foods to be as palatable to dogs and cats as conventional meat-based diets. Thus, dried yeast represents a viable ingredient option when needing a high-quality, palatable protein source for pet foods that may or may not contain animal-based ingredients.

Stool quality is another visual assessment pet owners use to determine whether their pet is tolerating a food. In addition, the degree to which a food is digested is used as an indicator of overall diet quality. In the current study, stool quality was equivalent or improved in dogs and cats receiving the Yeast vs. Control diets. For both dogs and cats, no significant differences in ATTD values for the Yeast and Control diet were observed for the measured nutrients, except for slightly lower ME for the Control diet when fed to cats. The high digestibility of the Yeast diet is consistent with previous studies that evaluated various yeast products in dogs. Reilly et al. (2021) examined the macronutrient ATTD in extruded canine diets containing 30% dried yeast. As reported by Reilly et al. (2021), a diet with 30% dried yeast diet was found to be highly digestible by adult dogs with ATTD values of 80.1% dry matter, 84.3% organic matter, 83.7% protein, and 97.9% fat. Although the ATTD for crude protein (83.7%) in the Reilly et al. (2021) study was lower than the current study (89.5%), they reported the ATTD values for crude protein were not statistically different for their control and 30% yeast-containing diets. The higher yeast inclusion in the Reilly et al. (2021) study may have contributed to the lower protein digestibility. This implies additional research is needed to investigate the dose–response of increasing dried yeast inclusion in pet foods on protein digestibility due to its potential effects on hindgut fermentation and fecal microbial protein contributions. Another study measured the macronutrient ATTD of dogs fed a diet containing 15% brewer’s yeast and reported apparent dry matter, organic matter, and crude protein digestibility values of 82.0%, 86.0%, and 86.2%, respectively, with no significant differences compared to a control diet without brewer’s yeast (Martins et al., 2014).

In the present study, all cats produced urine with pH and specific gravity values within the normal range for healthy adult cats. Although the specific gravity in the Control group (1.039 ± 0.0032) was significantly lower than the Yeast group (1.055 ± 0.0033), the values were still within the reference range for urine specific gravity (1.035 to 1.060), with values less than 1.035 being a possible indication of kidney disease (Bua et al., 2015). A limitation of this current study is the absence of baseline urinalysis values. As such, it is unknown if the lower urine specific gravity in the Control group is normal for this group of cats or if there was an effect of diet. In addition, water intake will affect urine specific gravity and it was not measured and may have differed between the two groups (Buckley et al., 2011). Regardless, the results provide support that the short-term feeding of the Yeast diet did not negatively impact urine pH or specific gravity as general indicators of urinary tract health in cats.

Dried yeast offers nutritional benefits, particularly due to its content of protein, fat, cell wall components, and fermentation metabolites. The crude protein content of the dried yeast used in the present study (51.8%; Table 1) is similar to protein values of other sources of dried whole yeast, such as brewer’s yeast (50.2% DMB) and sugarcane yeast (42.5%–45.5% DMB) as reported by Martins et al. (2013). The dried yeast used in the current study also had a similar crude fat value (3.3%; Table 1) than values previously reported for dried yeast, which has been shown to typically average about 6% fat (Rakowska et al., 2017). However, when the fat content of dried yeast was analyzed using acid hydrolysis prior to ether extraction, the values were reported to be higher (15.7%), likely due to acid hydrolysis liberating the ether-soluble components entrapped in the yeast cell wall (Reilly et al., 2020).

Dried yeast is a highly versatile ingredient that provides nutritional flexibility when used to formulate pet foods and treats. This flexibility is attributed to its balanced profile of essential amino acids and its inherently low levels of ash (3% to 4%) and calcium (0.02%). For pet food formulations, dried yeast provides a nutritional advantage over high-ash rendered animal protein meals. This is particularly important in formulas designed for large- and giant-breed puppies that require high-quality protein to support proper growth, but for which high dietary calcium can negatively impact musculoskeletal development. Large-breed puppies have a genetic predisposition for fast growth that can stress the developing skeletal structures resulting in skeletal malformations (Larsen, 2010). Excessive energy, which further accelerates the rate of growth, when coupled with excessive levels of calcium are also potentially detrimental to skeletal development of large-breed puppies (Goedegebuure and Hazewinkel, 1986; Larsen, 2010). As a high-quality protein that is low in calcium, dried yeast represents an ingredient that can be used by pet food formulators to achieve optimal nutrient profiles for large-breed puppies.

In conclusion, this study provides additional support regarding the acceptability and digestibility of dried whole yeast as an alternative protein for use in nutritionally complete and balanced dog and cat foods. At an inclusion level of 10%, the dried yeast significantly improved diet palatability in cats and demonstrated equivalent or better palatability in dogs. Digestibility was comparable between the test and control diets for all analyzed nutrients in both dogs and cats. Dried yeast is an alternative protein ingredient that meets the needs of discerning pet parents who are increasingly seeking alternative protein sources that are not derived from animal sources for both themselves and their pets.
